# Supplementary material for: Inhibition of miR-25 Ameliorates Cardiac Dysfunction and Fibrosis by Restoring Krüppel-like Factor 4 Expression
Source: Int J Mol Sci. 2023 Aug 4;24(15):12434. doi: 10.3390/ijms241512434 (PMC10418969; doi:10.3390/ijms241512434)
Supplement: Supplementary file 1 [file ijms-24-12434-s001.zip › ijms-2520554-supplementary.pdf]

The inhibition of miR-25 Ameliorates Cardiac Dysfunction and Fibrosis by Restoring Krüppel-like Factor 4 Expression

Supplementary Tables

Table S1. Echocardiographic parameter at 4 weeks tail vein injection with AAV9 EGFP and AAV9 miR-25 TuD

	sham (n=4)	AAV9-EGFP +Ang II (n=3)	AAV9-miR-25 TuD +Ang II (n=4)
IVSd(mm)	0.96±0.02	1.20±0.07††	1.10±0.01
LVIDd(mm)	4.52±0.09	4.74±0.10	4.62±0.09
LVPWd(mm)	0.98±0.01	1.12±0.05†	1.05±0.03
IVSs(mm)	1.60±0.04	1.81±0.09	1.82±0.06
LVIDs(mm)	3.13±0.11	3.66±0.13†	3.33±0.03
LVPWs(mm)	1.47±0.18	1.38±0.06	1.41±0.06
EF(%)	65.19±1.40	52.44±2.11††	60.83±1.37*
FS(%)	30.85±0.96	22.97±1.19††	27.98±0.89*
HR (BPM)	448.97±16.15	438.83±14.36	463.51±29.87

IVSD = interventricular septum, diastole (mm), LV = left ventricle, LVIDd = LV internal dimension, diastole (mm), LVPWd = LV posterior wall, diastole (mm), IVSs = interventricular septum, systole (mm), LVIDs = LV internal dimension, systole (mm), LVPWs = LV posterior wall, systole (mm), EF = ejection fraction (%), FS = fractional shortening (%), HR = heart rate (BPM), All data represent the mean±S.E.M of cardiac functional parameters. Statistical significance was determined by one-way ANOVA analysis of variance followed by the post hoc Tukey's multiple comparison test.

†p<0.05, ††p<0.01 vs sham

*p<0.05 vs AAV9-EGFP+Ang II

Table S2. Quantitative RT-PCR primer information

Gene		Sequence (5' to 3')
miR-25-3p	F	5'-CAT TGC ACT TGT CTC GGT CTG A- 3'
pri-miR-25	F	5'-CTC ACA GGA CAG CTG AAC ACC- 3'
	R	5'-CCC CCA CAT CTG CAG TGT TG- 3'
pre-miR-25	F	5'-CAG TGT TGA GAG GCG GAG ACT- 3'
	R	5'-GCA CTG TCA GAC CGA GAC AAG- 3'
β-MHC	F	5'-CCC AAG GAA AAG AAG CAC GTC- 3'
	R	5'-AGG TCA GCT GGA TAG CGA CAT C- 3'
ANF	F	5'-CGA GCA GCG GAT TGA ACT GT- 3'
	R	5′-TTG TGG TGA AGC CAC TCC TG- 3'
BNP	F	5'-CTC CTA CTA CGA GCT GAA CCA G- 3'
	R	5'-CCA GAA AGC TCA AAC TTG ACA GGC- 3'
IL-18	F	5'-GAC AAA AGA AAC CCG CCT G- 3'
	R	5'-ACA TCC TTC CAT CCT TCA CAG- 3'
IL-1β	F	5'-TCC AGG ATG AGG ACA TGA TGA GCA- 3'
	R	5'-GAA CGT CAC ACA CAC CAG CAG GTT A- 3'
IL-6	F	5'-CAA AGC CAG AGT CCT TCA GAG- 3'
	R	5'-GTC CTT AGC CAC TCC TTC TG- 3'
RANTES	F	5'-TGC AGA GGA CTC TGA GAC AGC- 3'
	R	5'-GAG TGG TGT CCG AGC CAT A- 3'
18s	F	5'-TAA CGA ACG AGA CTC TGG CAT-3'
	R	5'-CGG ACA TCT AAG GGC ATC ACAG-3'
U6		not available, Mir-X miRNA First-Strand Synthesis Kit components (Takara Bio Inc, Shiga, Japan)
